# Measurement and modeling of the adsorption isotherms of CH_4_ and C_2_H_6_ on shale samples

**DOI:** 10.1039/c9ra01432b

**Published:** 2019-05-03

**Authors:** Chao Li, Ligong Li, Tianhe Kang

**Affiliations:** Key Laboratory of In-situ Property-improving Mining of Ministry of Education, College of Mining Engineering, Taiyuan University of Technology Taiyuan Shanxi China 030024 kangtianhe@126.com +86-351-6014627

## Abstract

CH_4_ and C_2_H_6_ are two common components in shale gas. Adsorption isotherms of CH_4_, C_2_H_6_, and their binary mixtures on shale samples are significant for understanding the fundamental mechanisms of shale gas storage and the recovery of shale resources from shale reservoirs. In this study, the thermogravimetric method is applied to obtain the adsorption isotherms of CH_4_, C_2_H_6_ and their binary mixtures on two typical shale core samples. A simplified local density theory/Peng–Robinson equation of state (SLD-PR EOS) model is then applied to calculate the adsorption of CH_4_ and C_2_H_6_ on shale, and the efficiency of the SLD-PR EOS model is thus evaluated. The results show that C_2_H_6_ exhibits a higher adsorption capacity than CH_4_ on shale samples, indicating the greater affinity of C_2_H_6_ to organic shale. As the molar fraction of C_2_H_6_ increases in the CH_4_/C_2_H_6_ mixtures, the adsorption capacity of the gas mixtures increases, indicating the preferential adsorption of C_2_H_6_ on shale. Based on the predicted results from the SLD-PR EOS model, a reasonable agreement has been achieved with the measured adsorption isotherms of CH_4_ and C_2_H_6_, validating the reliability of the SLD-PR EOS model for predicting adsorption isotherms of CH_4_ and C_2_H_6_ on shale samples. In addition, the SLD-PR EOS model is more accurate in predicting the adsorption of CH_4_ on shale than that of C_2_H_6_. This study is expected to inspire a new strategy for predicting the adsorption of hydrocarbons on shale and to provide a basic understanding of competitive adsorption of gas mixtures in shale reservoirs.

## Abstract 2

CH_4_ and C_2_H_6_ are two common components in shale gas.

## Graphical abstract

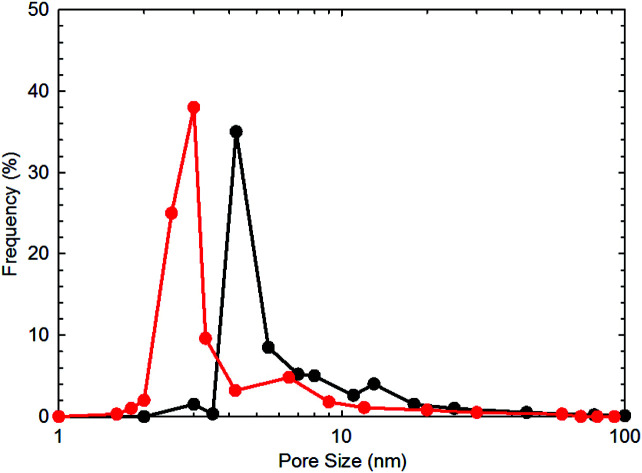

## 1. Introduction

Shale gas has been widely accepted as an important energy resource in recent years. Shale gas reservoirs possess some unique characteristics, such as extremely low permeability, rendering shale gas quite difficult to recover from such reservoirs. Compared to the conventional reservoirs, shale reservoirs generally have some unique characteristics, such as high organic content, which leads to more adsorption of hydrocarbons on shale.^1^ Understanding the adsorption behavior of shale gas is significant for estimating shale gas-in-place and the fundamental mechanisms of shale gas recovery.^2^

In recent years, the adsorption behavior of shale hydrocarbons has been extensively investigated. CH_4_ is the most abundant gas component in shale gas, which has been paid significant attention in the previous studies.^3–8^ Thermogravimetric analysis and the volumetric method are two commonly used approaches for measuring the adsorption isotherms of CH_4_ on shale.^9–14^ It is found that the thermogravimetric analysis method can measure the weight difference down to 1 μg; thereby, the thermogravimetric analysis method is more accurate than the volumetric method in determining the amount of adsorption on shale. Besides CH_4_, C_2_H_6_ also takes an important proportion in shale gas. CH_4_ and C_2_H_6_ generally show different adsorption capacity on shale rocks, the so-called competitive adsorption.^15^ Using thermogravimetric analysis method, Wang *et al.*^15^ measured the sorption isotherms of CH_4_ and C_2_H_6_ on shale and reveal the competitive adsorption behavior between both components on shale samples. However, their measurements were only conducted at temperature up to 333.15 K, which is not practical to shale reservoir conditions. Furthermore, to the best of our knowledge, studies regarding to C_2_H_6_ adsorption on shale is still scarce.

Extensive mathematical adsorption models have been proposed to match the adsorption of shale hydrocarbons on shale samples, including Langmuir model, the Brunauer–Emmett–Teller (BET) model, Dubinin–Astakhov (D–A), and Dubinin–Radushkevich (D–R) models.^16,17^ Compared to the Langmuir model and Brunauer–Emmett–Teller (BET) model, Dubinin–Astakhov (D–A) and Dubinin–Radushkevich (D–R) models are more accurate because they specifically consider the heterogeneous and hierarchical structures of shale cores.^18,19^ However, these aforementioned adsorption models are only a mathematical matching process without any physical meaning. Simplified local density (SLD) theory has been recently proposed to model the adsorption of hydrocarbons on shale; such model is more accurate than the conventional models due to its consideration of the pore surface–fluid interactions.^20^ In addition, most of the modeling works are conducted only for the CH_4_ adsorption, with less studies performed for C_2_H_6_. Here, one of the main motivations behind our efforts is to validate the SLD-PR EOS model in describing adsorption of light hydrocarbons, *i.e.*, CH_4_ and C_2_H_6_, on shale samples.

In this paper, the excess adsorption isotherms of CH_4_ and C_2_H_6_ and their binary gas mixtures are measured on two typical shale core samples using the thermogravimetric method. The adsorption of gas mixtures is compared with the adsorption of pure gases to reveal the occurrence of competitive adsorption under the shale reservoir conditions. The SLD-PR EOS model is then applied to predict the adsorption of gases on the shale samples and the effectiveness of the SLD-PR EOS model is then evaluated. The main objectives in this study are to understand the mechanisms of adsorption behavior of CH_4_ and C_2_H_6_ on shale samples and to evaluate the validity of SLD-PR EOS model in describing adsorption behavior of CH_4_ and C_2_H_6_ on shale. As a comprehensive study on gas adsorption behavior, the SLD-PR EOS model is the first time to be applied to model the adsorption isotherms of C_2_H_6_.

## 2. Experimental section

### 2.1 Materials

The gases, *i.e.*, CH_4_ and C_2_H_6_, used in this study have the purities of 99.90 wt% and 99.95 wt%, respectively. Thus, the uncertainty in the measurements is not caused from the impurity of gases. Two typical shale samples are retrieved from the depth of 1356 and 1437 m in the Longmaxi formation in the Sichuan Basin of China, where the reservoir temperature is approximately 343.15 K. In order to avoid the moisture in the air, the shale core samples are crushed into small particles and sealed in the zip-locked bags.

### 2.2 Characterization of shale core samples

In this work, the two shale samples are characterized to obtain the total organic carbon (TOC) and pore size distribution. TOC content is measured by a combustion elemental tester. First, H_2_SO_4_ is added into the shale particles to form a solution; O_2_ is then used to sparged the solution to remove the purgeable inorganic and organic carbon. The non-purgeable organic carbon is formed by CO_2_ in a combustion tube, which is then detected and used for the calculation of TOC content. The measured TOC content for each shale core sample is shown in Table 1.

**Table 1:** The measured TOC content and specific surface area of the typical shale core samples

Core sample	TOC content (wt%)	Dominate pore size (nm)	Specific surface area (m^2^ g^−1^)
#1	2.12	4.15	20.15
#2	2.53	3.00	25.32

**Fig. 1:**
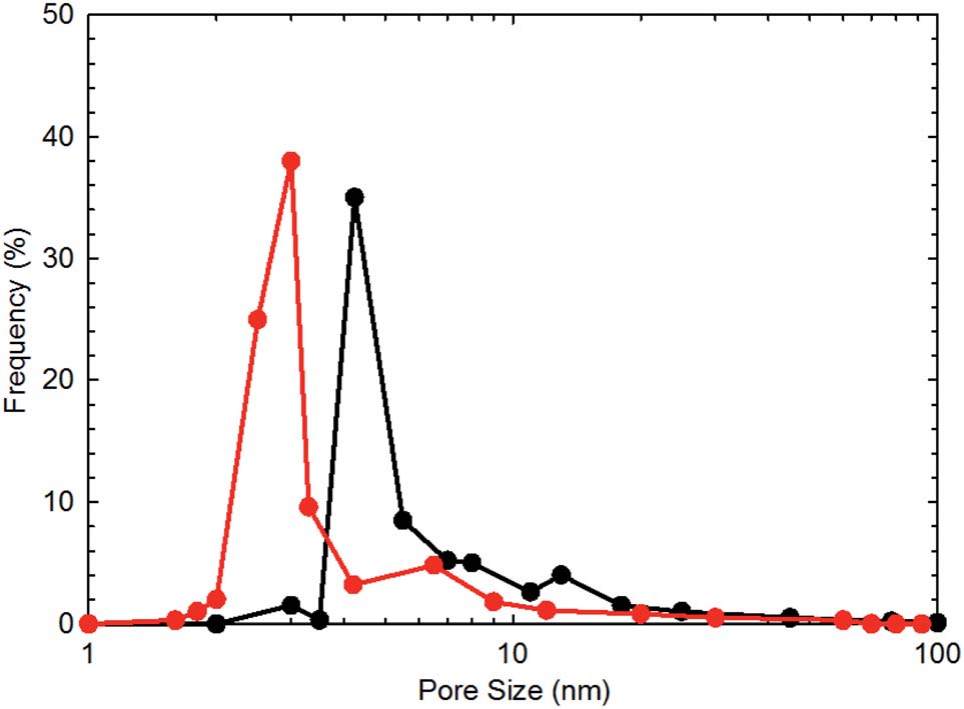
The measured pore size distribution for the two shale core samples.

To obtain the specific surface area and pore size distribution of the shale core samples, N_2_ adsorption/desorption tests are adopted. The gas sorption analyzer (Quantachrome, USA) is used for conducting the measurements by measuring the N_2_ adsorption/desorption at 77.0 K. The specific surface area is computed with the BET equation.^16^ The BET surface area for each shale sample are obtained with an accuracy of ±0.5%. The results of the BET surface area for each shale sample are shown in Table 1. Fig. 1 presents the measured pore size distribution for the two shale core samples. As shown in this figure, the two shale cores possess pores with the pore size falling in the nanoscale range. In addition, the dominant pore size for the two shale cores are 4.23 and 3.00 nm, respectively.

### 2.3 Measurements of adsorption isotherms

**Fig. 2:**
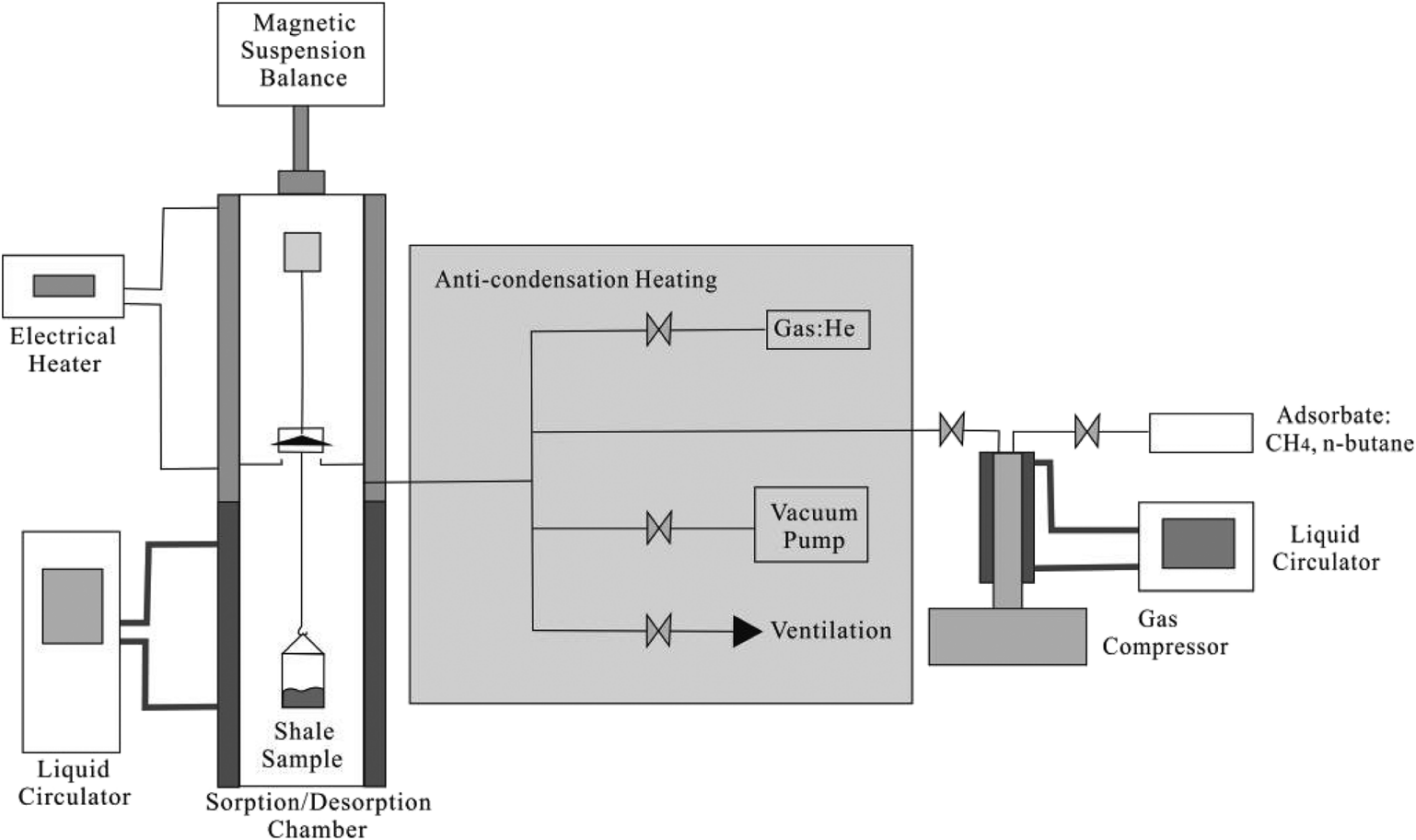
The schematic diagram of the experimental setup for measuring adsorption isotherms of CH_4_ and C_2_H_6_.

Adsorption isotherms of CH_4_ and C_2_H_6_ are measured by using an Intelligent Gravimetric Analyser (Nanjing Haohai Science Instruments and Apparatuses Limited Company, China). Fig. 2 presents the schematic diagram of the experimental setup for measuring adsorption isotherms of CH_4_ and C_2_H_6_. Before the isothermal measurements, the shale core particles are placed at 385.15 K and vacuumed for 12 hours for dehydration. The Gravimetric Analyser employs the thermogravimetric analysis approach for the measurement; this means the adsorption amount is obtained by calculating the weight change of shale sample. The mass of the empty sample container, *m*_c_, and its volume, *v*_c_, are first measured at the experimental temperature. The mass of shale sample, *m*_s_, and the sample volume, *v*_s_, are then measured by placing the shale sample into the adsorption chamber. Then, the adsorption chamber is filled with the adsorbent gas, *i.e.*, CH_4_ and C_2_H_6_ after vacuuming the sample chamber for 12 h at the experimental temperature. The pressure in the sample chamber increases gradually to the experimental value. Then, the apparent weight, Δ*m*, is measured at the given pressure and temperature until it reached stabilization,^2^

1 Δ*m* = *m*_c_ + *m*_s_ + *m*_a_ − (*v*_c_ + *v*_s_ + *v*_a_)*ρ*_b_

where *m*_a_ represents the mass of gas adsorbed on shale; *v*_a_ represents the adsorbed gas volume; *ρ* is the gas density in bulk; *m*_c_ and *v*_c_ represent the mass of the empty sample container and its volume, respectively; *m*_s_ and *v*_s_ represent the mass of shale sample and the sample volume, respectively.

The adsorbed mass can be calculated by,

2 *m*_a_ = Δ*m* − *m*_c_ − *m*_s_ + (*v*_c_ + *v*_s_ + *v*_a_)*ρ*_b_

Then the excess adsorbed mass, *m*_e_, can be calculated as,

3 *m*_e_ = *m*_a_ − *ρ*_b_*v*_a_ = Δ*m* − *m*_c_ − *m*_s_ + (*v*_c_ + *v*_s_)*ρ*_b_

## 3. Simplified local density/Peng–Robinson equation of state (SLD-PR EOS) model

The SLD-PR EOS model^21^ is applied to describe the CH_4_ and C_2_H_6_ adsorption on both shale core samples, while carbon-slit pores are employed to simulate the organic pores appeared in the shale samples. The SLD-PR EOS model can accurately calculate the fluid adsorption in nanopores by considering the fluid–fluid and fluid–solid surface interactions. Within the framework of the SLD-PR EOS model, the equation of state of CH_4_ and C_2_H_6_ employed the local-density approximation in obtaining the configurational energy of the adsorbed CH_4_ and C_2_H_6_. It is noted that the adsorbed CH_4_ and C_2_H_6_ distribute in-homogeneously in nanopores.^22^ Compared to molecular simulations, SLD/PR-EOS model considerably decreases the cost of computation.

Generally, three main assumptions are used in the SLD-PR EOS model,^22^

(1) Chemical potential of fluid at any point in nanopores is identical to the bulk chemical potential near the solid surface;

(2) Chemical potential of fluid in nanopores is the summation of fluid–fluid and fluid–surface potentials at adsorption equilibrium;

(3) Chemical potential from fluid-surface at any point is not influenced by molecules around this point.

At adsorption equilibrium, the chemical potential of CH_4_ and C_2_H_6_ at the position *z* is calculated by the potential summation due to the fluid–fluid and fluid–surface interactions; it equals to the chemical potential of CH_4_ and C_2_H_6_ in bulk.

4 *μ*(*z*) = *μ*_ff_(*z*) + *μ*_fs_(*z*) = *μ*_bulk_

where the subscript “ff” is the fluid–fluid interactions, “fs” is fluid–surface interactions, and “bulk” represents bulk CH_4_ and C_2_H_6_.

The bulk chemical potential of CH_4_ and C_2_H_6_ is expressed as a function of fugacity,5
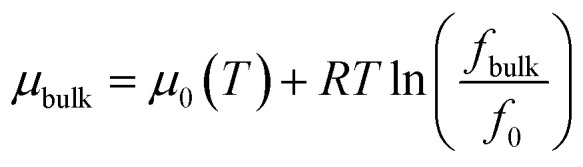
where *f*_bulk_ represents the bulk fugacity of CH_4_ and C_2_H_6_, *f*_0_ represents fugacity at a reference state, *μ*_bulk_ represents chemical potential in bulk; *μ*_0_ represents chemical potential at a reference state; *T* represents temperature; *R* represents ideal gas constant. The chemical potential of CH_4_ and C_2_H_6_ in nanopore from the CH_4_–CH_4_ and C_2_H_6_–C_2_H_6_ interactions is calculated as,6
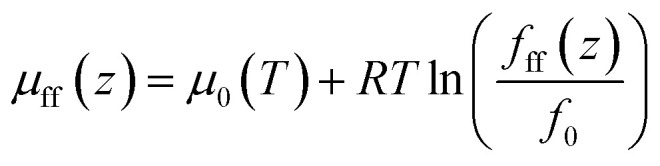
where *f*_ff_(*z*) represents the fugacity of CH_4_ and C_2_H_6_ at the position *z*; *f*_0_ represents fugacity at the same reference state as that in eqn (5).

The chemical potential of CH_4_ and C_2_H_6_ in nanopore from the CH_4_–solid surface interaction is calculated as,^21^

7 *μ*_fs_(*z*) = *N*_A_[*Ψ*^fs^(*z*) + *Ψ*^fs^(*L* − *z*)]

where *Ψ*^fs^(*z*) and *Ψ*^fs^(*L* − *z*) are interactions from the CH_4_–solid and C_2_H_6_–solid surface of a carbon-slit pore with a pore size of *L*; *N*_A_ is Avogadro's number. The CH_4_–solid and C_2_H_6_–solid surface interactions are approximated by the Lee's partially integrated 10–4 Lennard–Jones potential.^23^8
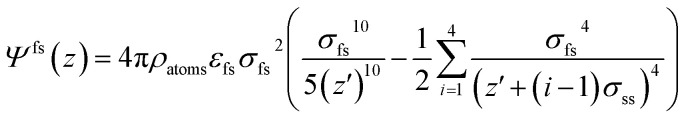
where *ρ*_atoms_ is the solid-atom density, 38.2 atoms per nm^2^;^24^*ε*_fs_ is the parameter from the CH_4_–solid surface and C_2_H_6_–solid surface interactions; *σ*_fs_ is the molecular diameters of CH_4_ and C_2_H_6_, which is computed by *σ*_fs_ = (*σ*_ff_ + *σ*_ss_)/2, where *σ*_ff_ and *σ*_ss_ represent molecular diameters of CH_4_ and C_2_H_6_ and the carbon-interplanar distance, respectively. The value of *σ*_ss_ is 0.355 nm for graphite; *z*′ represents the dummy coordinate, which is calculated as *z*′ = *z* + *σ*_ss_/2.

Substituting eqn (6)–(8) into eqn (4), the criterion for adsorption equilibrium is expressed as,9
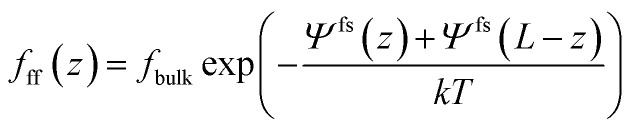
where *k* represents Boltzmann's constant, 1.38 × 10^−23^ J K^−1^; *T* represents absolute temperature.

The PR-EOS is used to calculate CH_4_–CH_4_ and C_2_H_6_–C_2_H_6_ interactions. The PR EOS can be given as a function of density (*ρ*),10
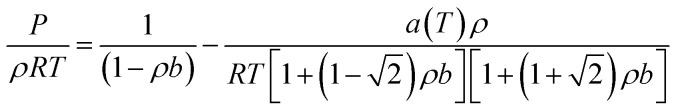
where11
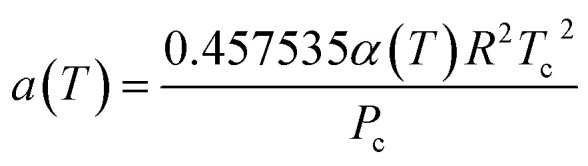
12
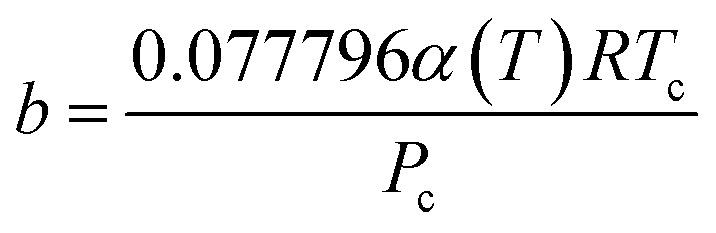


The *α*(*T*) term in eqn (11) is given with the following expression.^25^

13 *α*(*T*) = exp[(*A* + *BT*_r_)(1 − *T*_r_^*C*+*Dω*+*Eω*^2^^)]

where *A*, *B*, *C*, and *D* are correlation parameters, 2.0, 0.8145, 0.508, and −0.0467, respectively. The values of acentric factor (*ω*), the critical pressure (*P*_c_), the critical temperature (*T*_c_), and the molecular diameter for CH_4_ are 0.0113, 4.6 MPa, 190.56 K, and 0.3758 nm, respectively. Acentric factor (*ω*), the critical pressure (*P*_c_), the critical temperature (*T*_c_), and the molecular diameter for C_2_H_6_ are 0.0990, 4.9 MPa, 305.32 K, and 0.4000 nm, respectively.

In the PR-EOS, the fugacity of bulk CH_4_ and C_2_H_6_ are calculated as,14
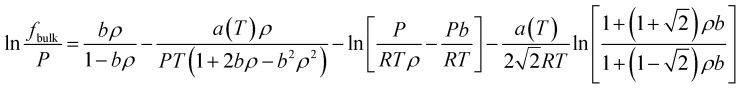
where *P* is the bulk pressure. With a similar analogy, fugacity of the adsorbate due to the CH_4_–CH_4_ and C_2_H_6_–C_2_H_6_ interactions is expressed as,15
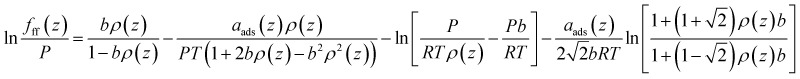
where *a*_ads_(*z*) is related with the position in the nanopore and the dimensionless pore width *L*/*σ*_ff_.^26^*a*_ads_(*z*) is obtained from Chen *et al.* (1997).^26^*ρ*(*z*) correlates with the position in carbon-slit pores, which represents the *in situ* gas density in nanopores.

In the PR EOS, covolume parameter *b* affects the local density of adsorbed CH_4_ and C_2_H_6_.^22^ To improve the predictive capacity of pure CH_4_ and C_2_H_6_ on carbon surface, Fitzgerald (2005)^27^ modified the covolume parameter *b*. To consider the repulsive interactions of the adsorbed CH_4_ and C_2_H_6_ at high pressure conditions, covolume parameter *b* is modified as,^27^

16 *b*_ads_ = *b*(1 + *Λ*_b_)

where *b*_ads_ is the modified covolume; *Λ*_b_ is the empirical correction for shale gases, ranging from −0.4 to 0.0.^22^ In our model, *Λ*_b_ is set as −0.20 for CH_4_ and C_2_H_6_. As a result, eqn (15) is expressed as,17
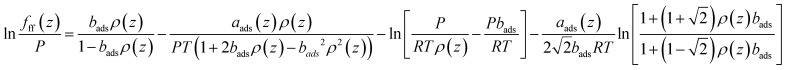


Density distribution of CH_4_ and C_2_H_6_ in nanopores can then be calculated by combining eqn (4) through (17). Within the SLD/PR-EOS model, the excess adsorption of CH_4_ and C_2_H_6_ is calculated as,18
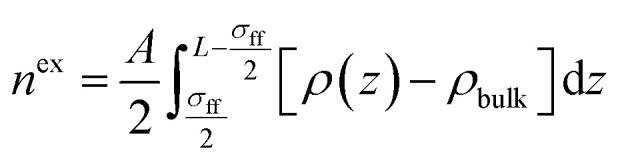
where *n*^ex^ represents the excess CH_4_ and C_2_H_6_ adsorption, which is calculated in moles per unit mass of adsorbent; *A* represents the total surface area of adsorbed CH_4_ and C_2_H_6_ on carbon surface. The lower limit of integration *σ*_ff_/2 is the center of the sphere-shaped CH_4_ and C_2_H_6_ molecules adsorbed on the pore surface, while the upper limit of integration *L* − (*σ*_ff_/2) is the center of CH_4_ and C_2_H_6_ molecules adsorbed on the pore surface of the other wall.

The average density (*ρ*_ave_) of CH_4_ and C_2_H_6_ in nanopores is expressed as,19
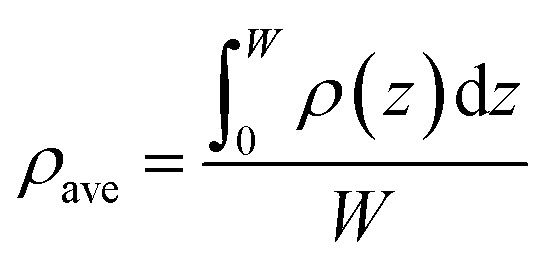
where *W* is the pore size of nanopore.

The SLD model applies the equation of state for CH_4_ and C_2_H_6_, which has been simplified with a local-density approximation in obtaining the configuration energy of the adsorbed CH_4_ and C_2_H_6_. The local-density approximation simplified the calculation for the long-range physical interactions, which is the difference from the conventional molecular simulation methods. This simplification renders the SLD model more efficient than the molecular simulation methods in calculating the confined fluid properties in nanopores, while it could be less accurate in describing some more complex molecules compared to the molecular simulation methods.

## 4. Results and discussion

### 4.1 Adsorption isotherms of CH_4_ and C_2_H_6_ on shale samples

**Fig. 3:**
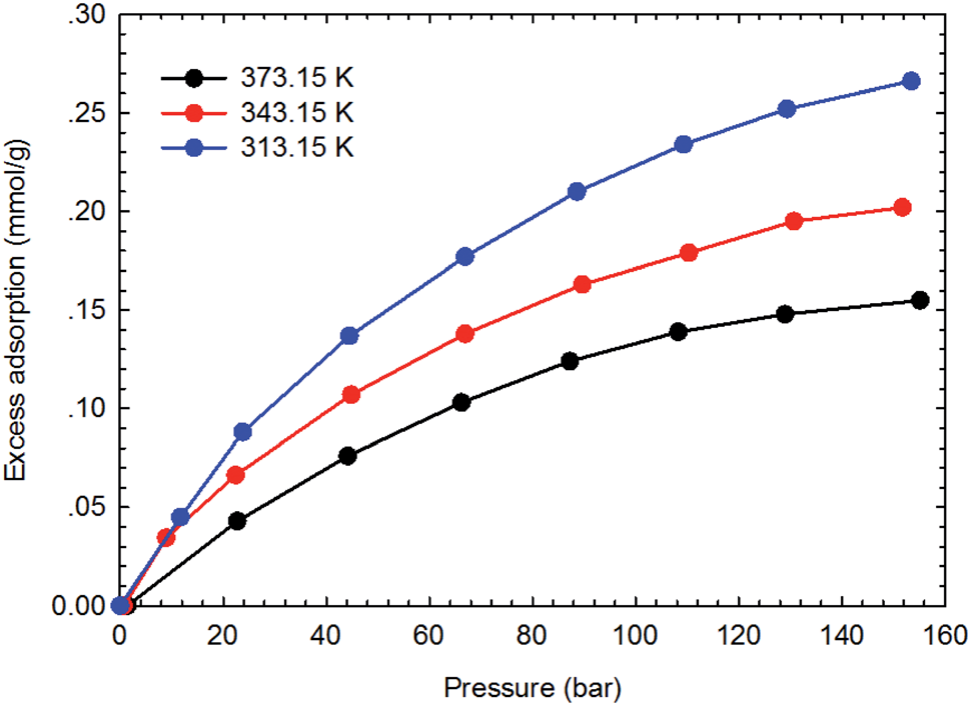
The measured excess adsorption of CH_4_ on the shale sample #1.

**Fig. 4:**
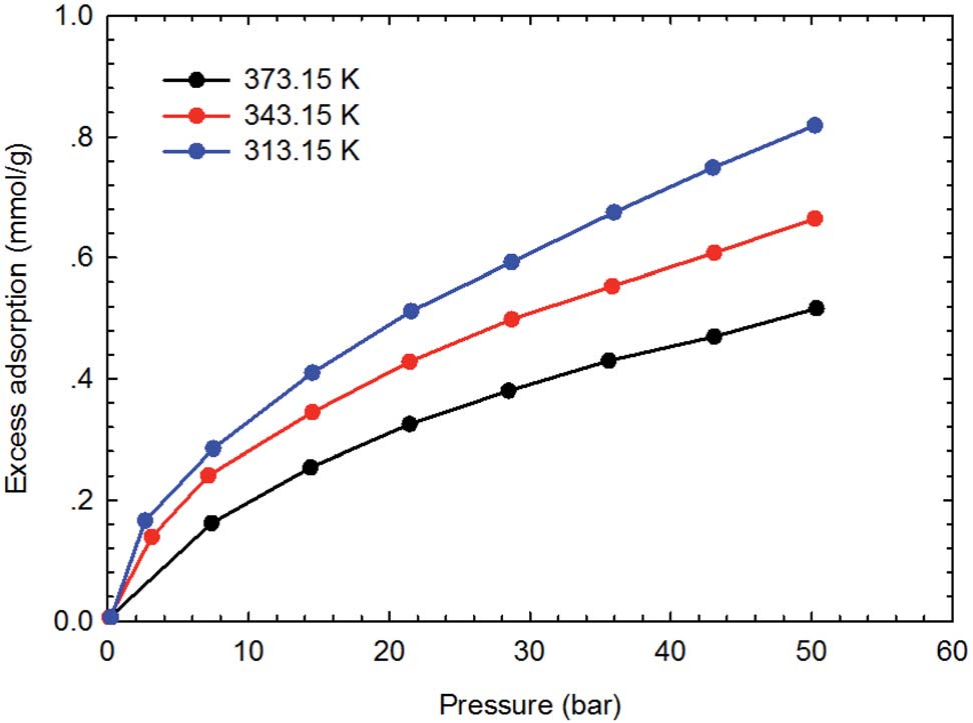
The measured excess adsorption of C_2_H_6_ on the shale sample #1.

**Fig. 5:**
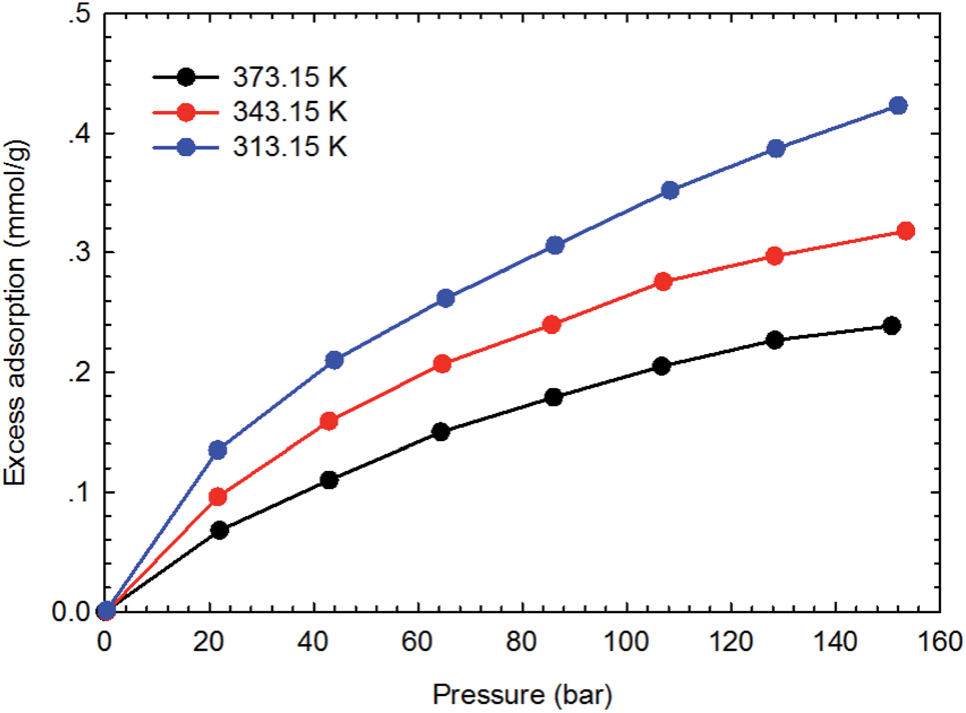
The measured excess adsorption of CH_4_ on the shale sample #1.

**Fig. 6:**
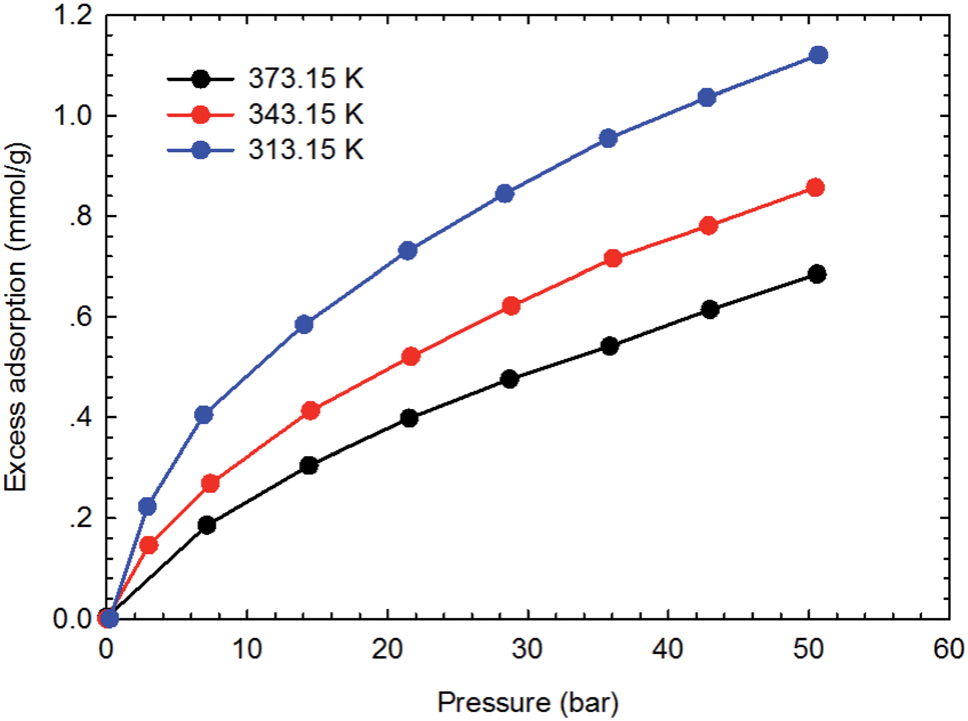
The measured excess adsorption of C_2_H_6_ on the shale sample #2.

Fig. 3–6 present the measured adsorption isotherms of CH_4_ and C_2_H_6_ on the two typical shale samples. It is observed that adsorption of CH_4_ and C_2_H_6_ is expected to be influenced by the system pressure and temperature; specifically, adsorption of CH_4_ and C_2_H_6_ increases as pressure increases but decreases as temperature increases. As for the same shale sample, C_2_H_6_ adsorption is significantly higher than that of CH_4_ at the same temperature and pressure conditions, indicating the more affinity of C_2_H_6_ to the organic shale. Compared with the shale sample #1, adsorption of CH_4_ and C_2_H_4_ on the shale sample #2 is much higher. Based on the characterization results for the two shale samples, the specific surface area and the total organic carbon content of shale sample #2 is significantly higher than that of the shale sample #1. The adsorption capacity of hydrocarbons on solid surface correlates with the physical properties of solid, such as surface area, and mineral-composition heterogeneity *etc.*^27^ Possibly, it is the main reason why the adsorption of CH_4_ and C_2_H_6_ on the shale sample #2 is stronger than that on the shale sample #1.

### 4.2 Competitive adsorption of CH_4_ and C_2_H_6_ on shale samples

**Fig. 7:**
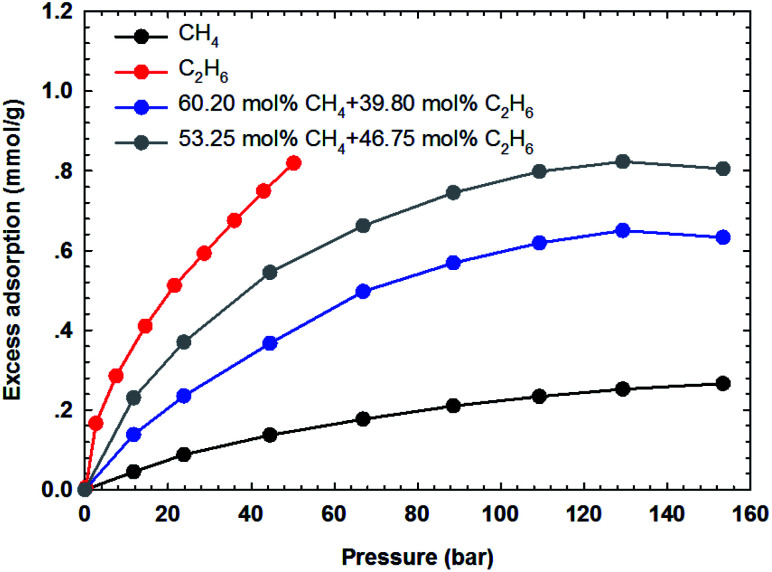
The measured excess adsorption of CH_4_–C_2_H_6_ mixtures on the shale sample #1 at 313.15 K.

**Fig. 8:**
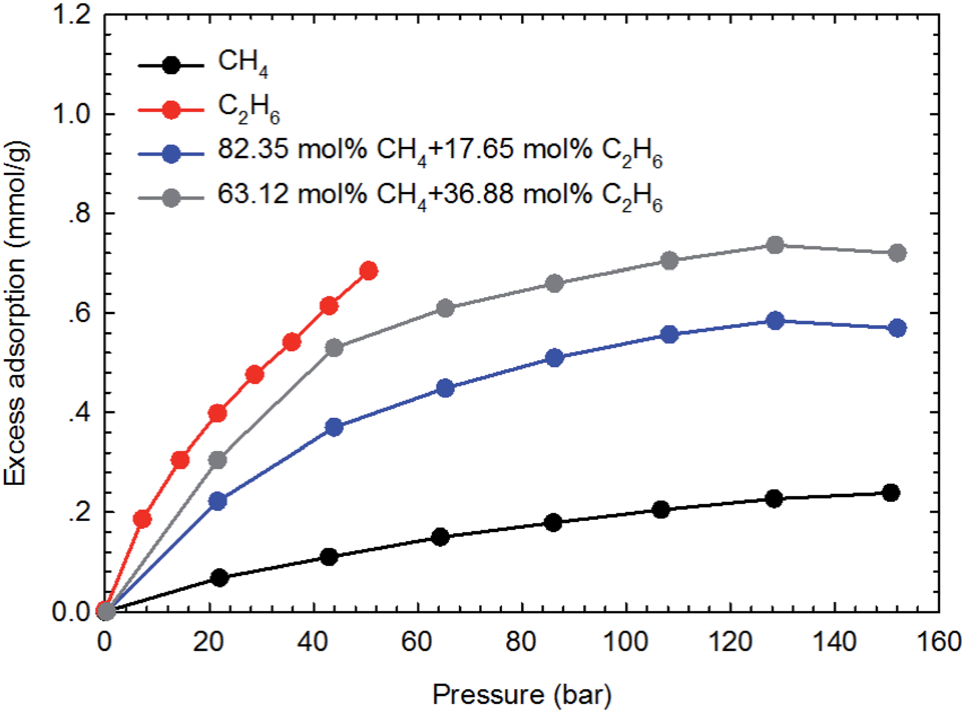
The measured excess adsorption of CH_4_–C_2_H_6_ mixtures on the shale sample #2 at 373.15 K.

The adsorption isotherms of the binary gas mixtures of CH_4_–C_2_H_6_ are measured on the two shale samples. In this work, four different gas compositions, *i.e.*, 60.20–39.80 mol%, 53.25–46.75 mol%, 82.35–17.65 mol%, and 63.12–36.88 mol% for CH_4_–C_2_H_6_ mixtures, are selected. The manner for isotherm measurements are conducted similarly to that adopted for the pure components. Fig. 7 and 8 show the measured adsorption isotherms for these gas mixtures. As for the two shale samples, the total excess adsorption of CH_4_–C_2_H_6_ mixtures increases as the molar concentration of C_2_H_6_ increases in the gas mixtures. It is possibly caused by the competitive adsorption between CH_4_ and C_2_H_6_ on the organic shale surface; C_2_H_6_ exhibits the preferential adsorption over CH_4_ on shale surface, resulting in a higher adsorption than CH_4_ but lower than C_2_H_6_. Additionally, we observe the maximum excess adsorption loading at about 130 bar for the four gas mixtures, while it tends to decrease beyond this pressure. However, this behavior is an exception for the pure gas adsorption isotherm under the studied conditions. The adsorption difference between gas mixtures and pure gases may be resulted from the interactions between two hydrocarbon species in the adsorption phase as well as in the free-gas phase.

### 4.3 SLD-PR EOS model for representing the adsorption of CH_4_ and C_2_H_6_

**Fig. 9:**
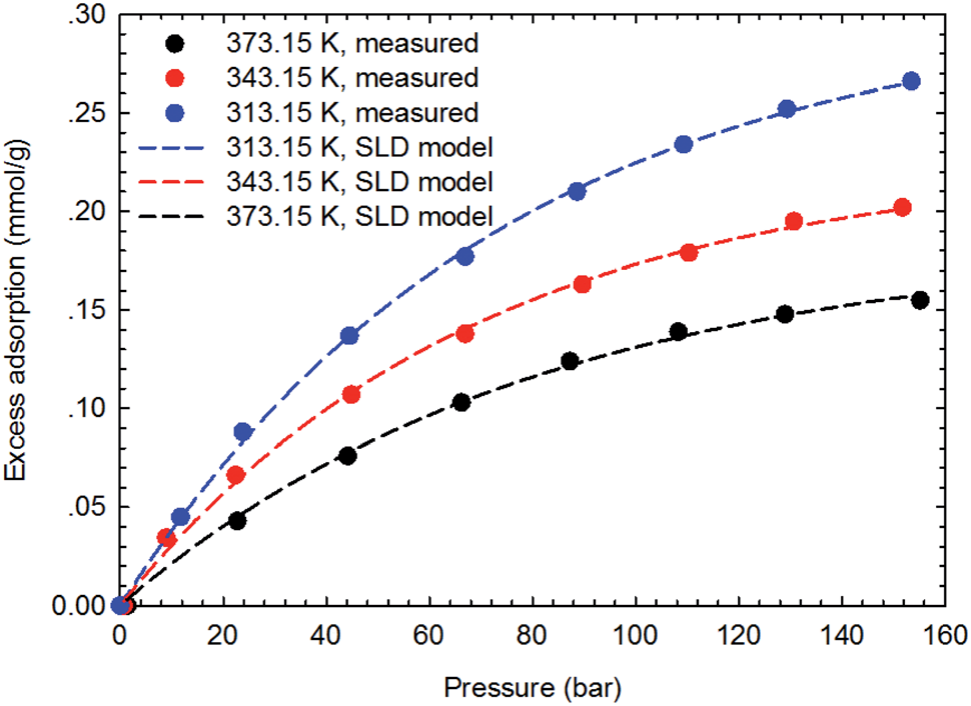
Curve fitting results with the measured excess adsorption of CH_4_ on the shale sample #1 using SLD-PR EOS model.

**Fig. 10:**
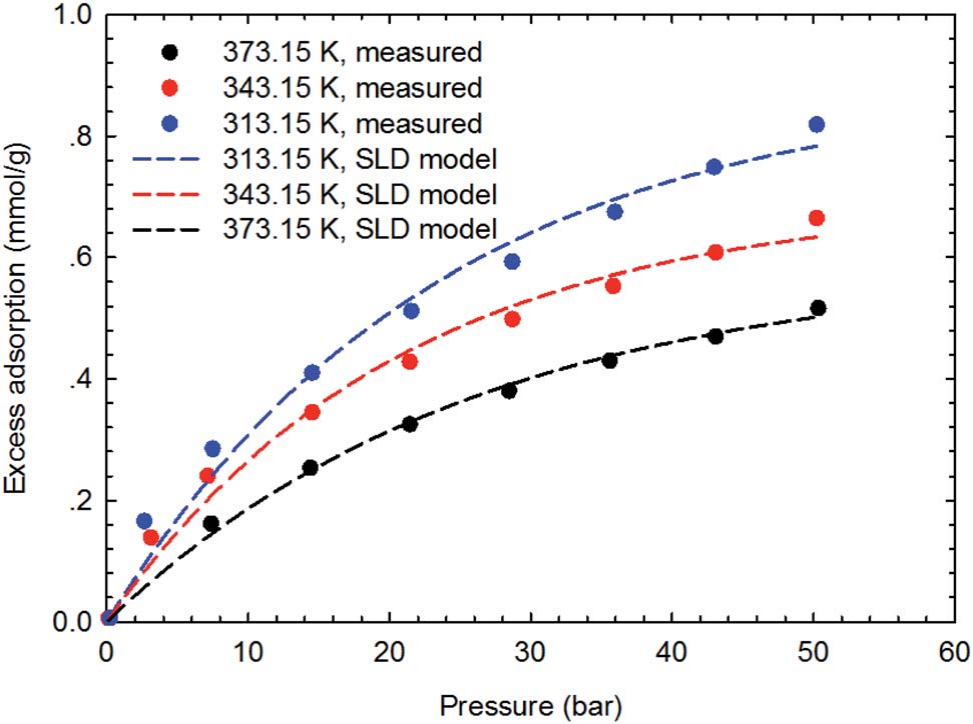
Curve fitting results with the measured excess adsorption of C_2_H_6_ on the shale sample #1 using SLD-PR EOS model.

**Fig. 11:**
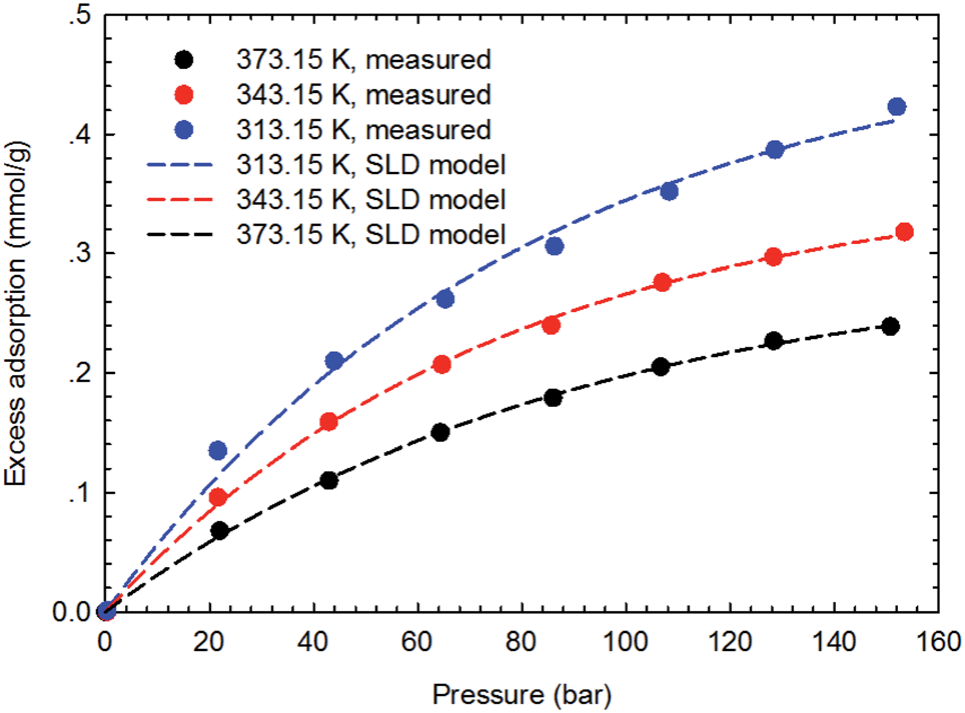
Curve fitting results with the measured excess adsorption of CH_4_ on the shale sample #2 using SLD-PR EOS model.

**Fig. 12:**
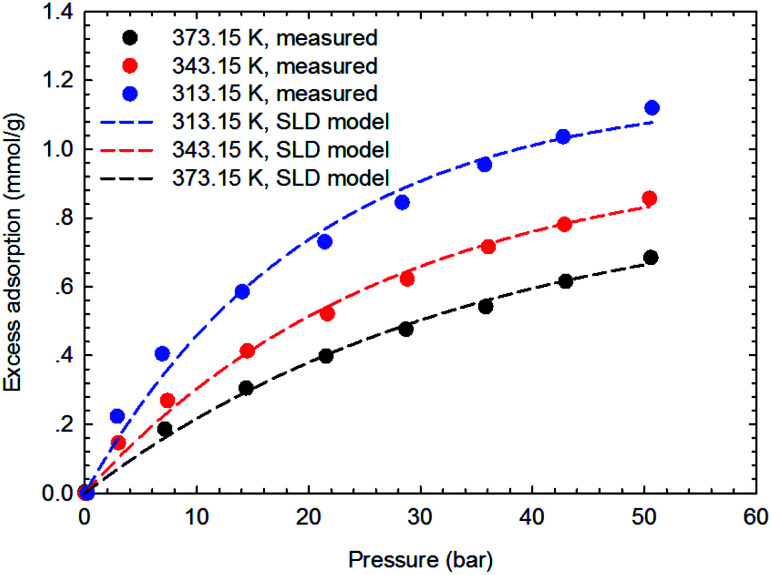
Curve fitting results with the measured excess adsorption of C_2_H_6_ on the shale sample #2 using SLD-PR EOS model.

The SLD-PR EOS model is applied to predict the adsorption of CH_4_ and C_2_H_6_ on shale samples, which are then applied to match the measured adsorption data. Specifically, two key parameters, *i.e.*, fluid-pore surface interaction energy (*ε*_fs_/*k*) and covolume correction parameter (*A*_b_), are adjusted in the SLD-PR EOS model to fit the measured excess adsorption. Table 2 shows the adjusted parameters in the SLD-PR EOS model. Fig. 9–12 present the comparison results between the measured excess adsorption and the predicted excess adsorption of CH_4_ and C_2_H_6_ from the SLD-PR EOS model. We observe that the SLD-PR EOS model can reasonably represent the measured excess CH_4_ and C_2_H_6_ adsorption on the two shale samples. In addition, compared to CH_4_, we observe that the SLD-PR EOS model is less accurate for predicting C_2_H_6_ adsorption.

**Table 2:** The key parameters input in the SLD-PR EOS model for predicting the gas adsorption

Gas sample	Core sample	Temperature (K)	*L* (nm)	*ε*_fs_/*k* (K)	*A*_b_	*A* (m^2^ g^−1^)
CH_4_	#1	313.15	4.15	51.2	0.026	20.15
		343.15	4.15	54.5	0.055	20.15
		373.15	4.15	57.6	0.051	20.15
	#2	313.15	3.00	63.2	0.132	25.32
		343.15	3.00	66.3	0.136	25.32
		373.15	3.00	64.5	0.127	25.32
C_2_H_6_	#1	313.15	4.15	53.5	0.035	20.15
		343.15	4.15	56.8	0.056	20.15
		373.15	4.15	59.3	0.032	20.15
	#2	313.15	3.00	67.1	0.125	25.32
		343.15	3.00	68.5	0.121	25.32
		373.15	3.00	67.2	0.120	25.32

Based on the comparison results, the absolute relative error of the calculated adsorption of CH_4_ and C_2_H_6_ are calculated from the measured excess adsorption. The absolute relative error is calculated as,20
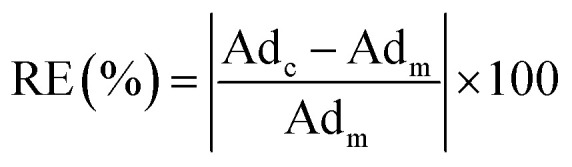
where RE represents the absolute relative error, %; Ad_c_ represents the calculated adsorption on shale surface, mmol g; Ad_m_ represents the measured excess adsorption on shale surface, mmol g^−1^.

**Fig. 13:**
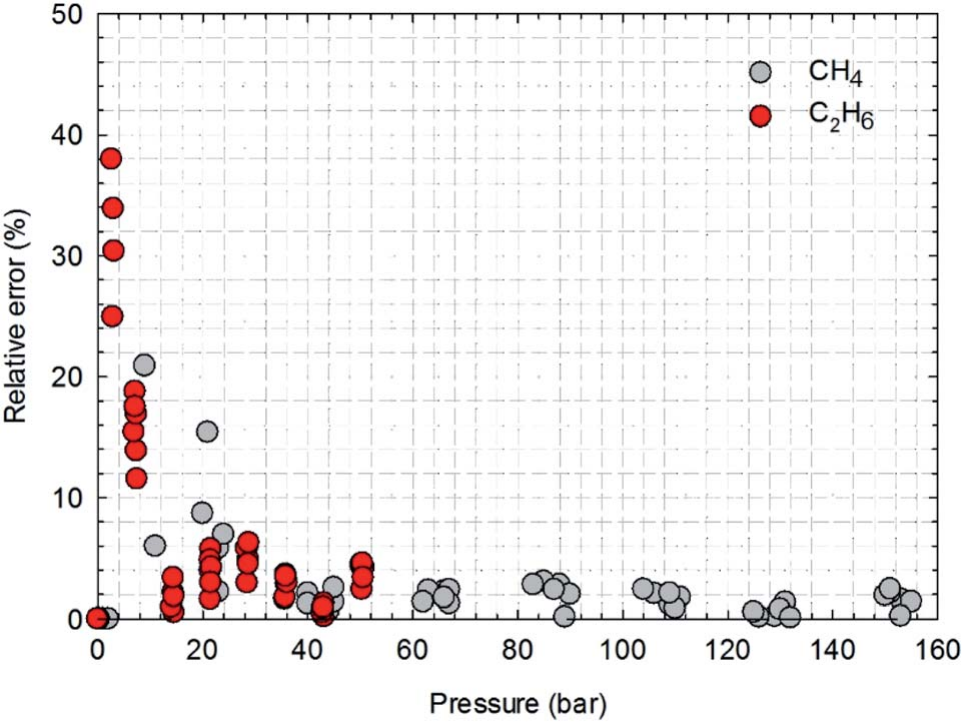
The calculated absolute relative error for CH_4_ and C_2_H_6_ at various pressure conditions.

Fig. 13 presents the calculated absolute relative error for CH_4_ and C_2_H_6_ at various pressure conditions. We observe a higher absolute relative error at lower pressures for both CH_4_ and C_2_H_6_, which, specially, can be as high as 40% for C_2_H_6_, while the absolute relative error decreases as pressure increases. It suggests the SLD-PR EOS model is not accurate in predicting adsorption of CH_4_ and C_2_H_6_ on shale samples at low pressure conditions. In addition, compared with CH_4_, a much higher absolute relative error is observed for C_2_H_6_, indicating that the SLD-PR EOS model may not be suitable for the prediction of the adsorption of heavier hydrocarbon species.

## 5. Conclusions

In this work, the excess adsorption isotherms of CH_4_, C_2_H_6_ and their binary gas mixtures are measured on two typical shale core samples using thermogravimetric method. The adsorption of gas mixtures is compared with that of pure gases to reveal the behavior of competitive adsorption under the shale reservoir conditions. The SLD-PR EOS model is then applied for predicting the adsorption of CH_4_ and C_2_H_6_ on both shale samples to evaluate its efficiency in predicting the adsorption of shale hydrocarbons. The detailed conclusions can be drawn as below:

(•) C_2_H_6_ has higher adsorption capacity than CH_4_ on the two shale samples under the same conditions; it suggests the more affinity of C_2_H_6_ on the organic shale;

(•) As observed from the measured adsorption isotherms of CH_4_–C_2_H_6_ mixtures, as the molar fraction of C_2_H_6_ in CH_4_–C_2_H_6_ mixtures increases, adsorption of the gas mixture increases, indicating the preferential adsorption of C_2_H_6_ on shale.

(•) Based on the predicted results from the SLD-PR EOS model, a reasonable agreement has been achieved with the measured adsorption isotherms, indicating the accuracy of the SLD-PR EOS model in predicting the gas adsorption on shale samples. In addition, compared with C_2_H_4_, the SLD-PR EOS model is more accurate in predicting adsorption of CH_4_ on shale.

This study proposes the SLD-PR EOS model for the prediction of gas adsorption on shale samples; in addition, it may provide a basic understanding of the competitive adsorption of hydrocarbons in shale reservoirs. To our knowledge, the adsorption measurements of gas mixtures on typical shale samples are presented for the first time. However, future works should be supplemented to our study. Besides CH_4_ and C_2_H_6_, some other heavier hydrocarbons, such as *n*C_3_H_8_, *n*C_4_H_10_, may also be an important component in shale gas. Thereby, future works are suggested to measure the adsorption/desorption isotherms of the heavier hydrocarbons on shale. In addition, in our work, we measure the adsorption of C_2_H_6_ at pressures as high as 60 bar based on the saturated vapor pressure of C_2_H_6_ at given temperature. New experimental setups should be designed to achieve the adsorption measurement at pressures as close as the shale reservoir conditions.

## Conflicts of interest

There are no conflicts to declare.

## Supplementary Material
